# Mapping of the Sequences Directing Localization of the *Drosophila* Germ Cell-Expressed Protein (GCE)

**DOI:** 10.1371/journal.pone.0133307

**Published:** 2015-07-17

**Authors:** Beata Greb-Markiewicz, Daria Sadowska, Natalia Surgut, Jakub Godlewski, Mirosław Zarębski, Andrzej Ożyhar

**Affiliations:** 1 Department of Biochemistry, Faculty of Chemistry, Wrocław University of Technology, Wrocław, Poland; 2 Department of Neurosurgery, Brigham and Woman's Hospital, Harvard Medical School, Harvard Institute of Medicine, Boston, Massachusetts, United States of America; 3 Department of Cell Biophysics, Faculty of Biochemistry, Biophysics and Biotechnology, Jagiellonian University, Kraków, Poland; University of Kentucky, UNITED STATES

## Abstract

*Drosophila melanogaster* germ cell-expressed protein (GCE) belongs to the family of bHLH-PAS transcription factors that are the regulators of gene expression networks that determine many physiological and developmental processes. GCE is a homolog of *D*. *melanogaster* methoprene tolerant protein (MET), a key mediator of anti-metamorphic signaling in insects and the putative juvenile hormone receptor. Recently, it has been shown that the functions of MET and GCE are only partially redundant and tissue specific. The ability of bHLH-PAS proteins to fulfill their function depends on proper intracellular trafficking, determined by specific sequences, i.e. the nuclear localization signal (NLS) and the nuclear export signal (NES). Nevertheless, until now no data has been published on the GCE intracellular shuttling and localization signals. We performed confocal microscopy analysis of the subcellular distribution of GCE fused with yellow fluorescent protein (YFP) and YFP-GCE derivatives which allowed us to characterize the details of the subcellular traffic of this protein. We demonstrate that GCE possess specific pattern of localization signals, only partially consistent with presented previously for MET. The presence of a strong NLS in the C-terminal part of GCE, seems to be unique and important feature of this protein. The intracellular localization of GCE appears to be determined by the NLSs localized in PAS-B domain and C-terminal fragment of GCE, and NESs localized in PAS-A, PAS-B domains and C-terminal fragment of GCE. NLSs activity can be modified by juvenile hormone (JH) and other partners, likely 14-3-3 proteins.

## Introduction

*Drosophila melanogaster* has become an important model to study a diverse range of biological processes including understanding how genes direct the development of an embryo from a single cell to a mature multicellular organism [1]. The growth and development of insects is controlled by two hormones: the steroid 20-hydroxyecdysone (20E) and the sesquiterpenoid juvenile hormone (JH) [2]. Both pathways interact to mediate insect development, but the detailed mechanism of these interactions has not yet been fully elucidated [3,4]. The simultaneous presence of both 20E and JH leads to larval-larval molting, while a lack of JH initiates metamorphosis. Additionally, JH is responsible for regulating maturation of the reproductive system. Despite its importance in the developmental processes of insects, the receptor for JH was unknown for a long time [5,6]. Wilson and Fabian [7] discovered the *Methoprene-tolerant* (*Met*) gene while screening for mutants resistant to the JH analog methoprene, used as an insecticide. The genomic localization of the *Met* gene and the homology of the MET protein to the family of bHLH-PAS transcription factors was subsequently discovered [8]. MET has gained the status of being the putative JH receptor, because of its ability to bind JH with nanomolar affinity and to initiate transcriptional activity in a JH-dependent manner [9,10]. However, the fact that the *Drosophila* Met-null mutant is fully viable undermined the credibility of the hypothesis that MET functions as a JH receptor [7]. Later on, the bHLH-PAS paralog of MET, the germ cell- expressed protein (GCE) was discovered [11]. There was also a recent finding that partial functional redundancy prevents the mortality of mutant flies [12,13].

It was documented that MET is capable of forming both homodimers and heterodimers with GCE as a partner protein in the absence of JH [14]. In contrast to MET, the overexpression of GCE does not cause mortality [12]. Studies on GCE have revealed that the expression of MET is more abundant [13], but that GCE binds JH with higher activity [10]. The activation of *E75A*, a JH target gene, requires GCE but not MET, providing proof that GCE is not merely a MET substitute [15] and opening the possibility of intricate MET/GCE-specific functions. Dubrovsky *et al*. found evidence that the orphan nuclear receptor FTZ-F1 is an essential component of JH signaling by interaction with putative JH receptors MET and GCE [15]. The course of evolution of both genes across the *Drosophila* genus suggests that *Met* appeared as a product of the duplication of the *Gce*-like ancestor gene during early dipteran evolution [16]. It has been shown that MET is able to interact with proteins involved in the signal transduction of ecdysteroids: ecdysteroid receptor (EcR) and ultraspiracle (USP) but also with 39 kDa FK506-binding nuclear protein (FKBP39), calponin-like protein Chd64 (Chd64) [3,17], steroid receptor co-activator (SRC) [18,19] and cycle protein [20] as well. MET is a component of a protein complex that coordinates the crosstalk between JH and 20E signaling pathways [3,16,21]. MET and GCE induced programmed cell death (PCD), and this activity may be suppressed by methoprene [22]. The published data on the role of JH reception, including review reports [23,24,25], focused primarily on the function of MET and left the function of GCE largely unknown [24].

The family of bHLH-PAS transcription factors includes proteins that are critical regulators of the gene expression network responsible for many essential physiological and developmental processes in invertebrates [26,27]. The ability to localize and translocate these proteins to specific cellular compartments is fundamental to the organization and functioning of all living cells. For a number of transcription factors, translocation from the cytoplasm to the nucleus is an important event that enables the transcription factor to recruit co-activators [28]. Recently He *et al*. [29] reported that Hsp83 facilitates the JH induced nuclear import of MET in *D*. *melanogaster* larval fat body cells. The nuclear transport of proteins is usually mediated by a family of transport receptors known as karyopherins, which form a transport complex after binding to proteins as a result of the recognition of the nuclear localization signal (NLS) for nuclear import or the nuclear export signal (NES) for export [30]. The best characterized transport signal is the classical NLS (cNLS) for nuclear protein import, which consists of either one (monopartite) or two (bipartite) stretches of basic amino acids [31]. The most common characterized NES consists of a non-conserved motif encompassing hydrophobic residues and is leucine-rich [30]. The high degree of homology between the GCE and MET proteins [32] raises the possibility that the trafficking of GCE in the cell is mediated by some of the localization signals identified for MET [33]. However, the sequence homology applies only to the bHLH (78%) and PAS domains (PAS-A 68%, PAS-B 86%) [11]. The identification of the NLS and NES signals is fundamental in being able to understand the intracellular signaling role of the GCE protein. In order to better determine the presence of NLS and NES signals in the GCE protein we decided to use mammalian cells which do not produce juvenile hormone and 20-hydroxyecdysone. This cells are also devoid of some insect cell-specific endogenous proteins like MET and the EcR/USP complex, which could influence results obtained in insect cells. Mammalian cells were used successfully to analyze the subcellular trafficking of MET [33], and transcriptional response of EcR [6] and MET/GCE to JH [10,34]. We investigated the subcellular distribution of GCE in living cells using a yellow fluorescent protein (YFP) label.

Here we shown that GCE has a specific pattern of signals that are only partially consistent with those previously presented for MET. The homology of GCE and MET with regards to NLS and NES activity occurs only in the PAS domains. We demonstrate the presence of a strong NLS in the C-terminal fragment of GCE, which seems to be a unique and important feature of this protein.

## Materials and Methods

### Plasmid construction

GCE cDNA from *D*. *melanogaster* was a kind gift from Prof. Thomas G. Wilson (Department of Entomology, Ohio State University, USA) [13]. We numbered amino acids residues of GCE from 1 to 689 in accordance with papers previously published [35,36]. According to personal communication with A. Baumann (University of Tennessee), GCE used in our study and its longer variant [16] deposited in UniprotKB database as Q9VXW7 show similar function when expressed in transgenic *D*. *melanogaster*.

Full-length cDNA, encoding amino acid residues 1–689, was amplified by PCR and cloned into the *Hind*III and *Sma*I restriction sites of the MCS of the pEYFP-C1 vector. Deletion mutants of GCE were cloned analogically in the pEYFP-C1 vector. DNA constructs: YFP-GCE/S462A/S670A, YFP-GCE/S462A, YFP-GCE/S670A, YFP-GCE430-572/S462A, YFP-GCE573-689/S670A, YFP-GCE391-572/S462A, YFP-GCE391-689/S462A, YFP-GCE391-689/S670A and YFP-GCE391-689/S462A/S670A were analogically obtained for the full-length and deletion mutants, and for the PCR reaction templates GCE/S462A/S670A, GCE/S462A or GCE/S670A were used. Mutations were introduced using the QuikChange Site-Directed Mutagenesis Kit (Stratagene), according to the manufacturer’s instructions. The point mutants, GCE69-429/K356A/K360A/H362A, GCE/K582A/K585A, GCE/S462A, K582A/K585A, GCE/K582A/K585A/S670A, GCE/S462A/K579A/K582A/S670A, GCE391-689/K582A/K585A, GCE391-689/S462A/K582A/K585A, GCE391-689/K582A/K585A/S670A, and GCE391-689/S462A/K582A/K585A/S670A, were obtained by the PCR-Mediated Site-Directed Mutagenesis as described by Ko and Ma [37], where GCE, GCE/S462A/S670A, GCE/S462A or GCE/S670A were used as a template and cloned with *Lgu*I, *Hind*III and *Sma*I restriction enzymes. All constructs were verified by DNA sequencing.

### Cell culture and DNA transfection

African green monkey kidney fibroblasts COS-7 (ATCC CRL-1651) and human embryonic kidney HEK293 (Sigma) cells were maintained in Dulbecco’s modified Eagle medium (DMEM) supplemented with 1% non-essential amino acids (Gibco/Invitrogen), 1 mM sodium pyruvate and 2% glutamine (Gibco/Invitrogen). The medium was supplemented with 10% fetal calf serum (FCS). Cells were grown at 37°C in a 95% air/5% CO_2_ atmosphere. Cells were transfected with 3 μg DNA/150 000 cells using jetPEI (Polyplus-transfection SA), according to the manufacturer’s instructions. Juvenile hormone III (JHIII) (Sigma) was dissolved in dimethylsulfoxide (DMSO) (Sigma) to a concentration of 10^−3^ M and added to the medium during transfection to a final concentration of 10^−6^ M. The control cells received an equal volume of DMSO.

### Confocal fluorescence microscopy

Before conducting the imaging experiments, cells were plated on 0.17-mm-thick round glass coverslips (Menzel) submerged in a culture medium in 2-cm-diammeter Petri dishes. Prior to the microscopy experiment (20–24 h after transfection), coverslips with cell cultures were transferred onto a steel holder and mounted on a microscope stage. The standard culture medium was replaced by DMEM/F12 without phenol red, buffered with 15 mM HEPES (Sigma), suplemented with 1% FBS (Sigma). During microscopy, the temperature of the cell culture was maintained at 37°C by microincubator (Life Imaging Services Box & Cube). Images of fluorescently labeled proteins were acquired using the Leica TCS SP5 II confocal system equipped with an argon laser and a 63xOil (NA: 1,4) objective lens. YFP was excited using 514 nm light and the emitted fluorescence was observed at range 525–600 nm. Images are presented for typical cells with phenotype characteristic for more than 95% of the observed cells population.

### *In silico* analysis of the GCE sequence

To predict the secondary structure of GCE we used PSIPRED (Protein Structure Prediction Server) [38], http://www.psipred.net/psiform.html. To predict regions in GCE that do not assume unique three-dimensional structures (intrinsically disordered regions, IDRs) [39] we used Pondr, http://www.pondr.com/, [40–42]. To predict the domain architecture of GCE we used SMART (Simple modular architecture tool) [43], http://smart.embl-heidelberg.de/, PROSITE (a database of protein families and domains), http://expasy.org/tools/scanprosite/, and Protein Model Portal (PMP), http://www.proteinmodelportal.org/. The sequence alignments were obtained by CLUSTAL_X [44], http://www.clustal.org/. Predictions for the potential NLS signals were performed by NucPred [45], http://www.sbc.su.se/~maccallr/nucpred/, PSORTII [46], http://www.psort.org/, cNLS Mapper http://nls-mapper.iab.keio.ac.jp/cgi-bin/NLS_Mapper_y.cgi. Predictions of the potential NES signals were performed by the NetNes 1.1 server [47], http://www.cbs.dtu.dk/services/NetNES/, and ValidNES [48], http://validness.ym.edu.tw. Scansite http://scansite.mit.edu was used to predict potential 14-3-3 binding sites [49] along with the Eukaryotic Linear Motif resource for Functional Sites in Proteins (ELM) [50], http://elm.eu.org/. Potential phosphorylation sites were predicted with the Disorder-Enhanced Phosphorylation Site Predictor (Disphos 1.3), http://www.dabi.temple.edu/disphos/, [51–55] and NetPhos 2.0 [56], http://www.cbs.dtu.dk/services/NetPhos/.

## Results

### Searching for nuclear import and export signals in different parts of GCE

We performed a set of experiments to identify NLS and NES motifs in GCE, using full-length GCE and a series of deletion mutants tagged with YFP. To ensure the functionality of the structural motifs within the GCE sequence, truncated regions were designed using the results of NLS and NES prediction methods, the secondary structure motifs of GCE, the alignment of the GCE sequence with MET and predictions of GCE domains (data not shown). The subcellular localization of the expressed proteins was analyzed by confocal microscopy 20-24h after the transfection of COS-7 cells. Then, western blot analysis was performed to prove the expression of YFP tagged proteins (data not shown).

**Fig 1:**
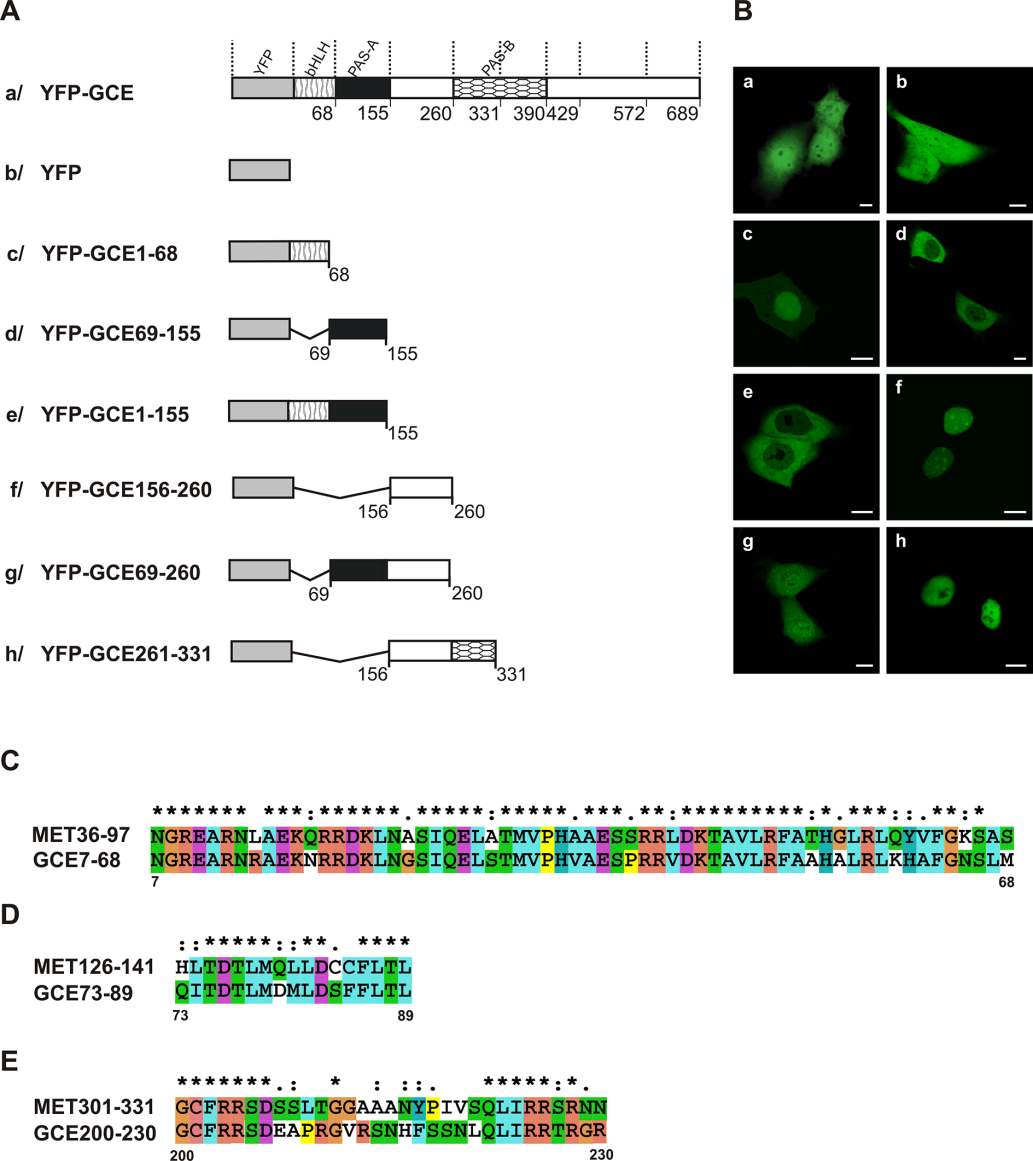
Subcellular distribution of the GCE mutants. Experiments identifying NLS and NES motifs in GCE, using full-length GCE and a series of deletion mutants tagged with YFP revealed differentiated localization of truncated proteins. The subcellular localization of the expressed proteins was analyzed by confocal microscopy 20-24h after the transfection of COS-7 cells. (A) Schematic representation of the GCE and deletion mutants. Regions of GCE are depicted using different patterns. The length of each domain in the diagram is arbitrary. (B) Representative images (single confocal plane) of the subcellular distribution of the GCE derivatives. Bar, 10 μm. (C) ClustalX alignment of the MET36-97/GCE7-68 encompassing bHLH domain. (D) ClustalX alignment of the MET126-141/GCE73-89 encompassing NES in PAS-A domain. (E) ClustalX alignment of the MET301-331/GCE200-230 from area linking PAS-A with PAS-B.

**Fig 2:**
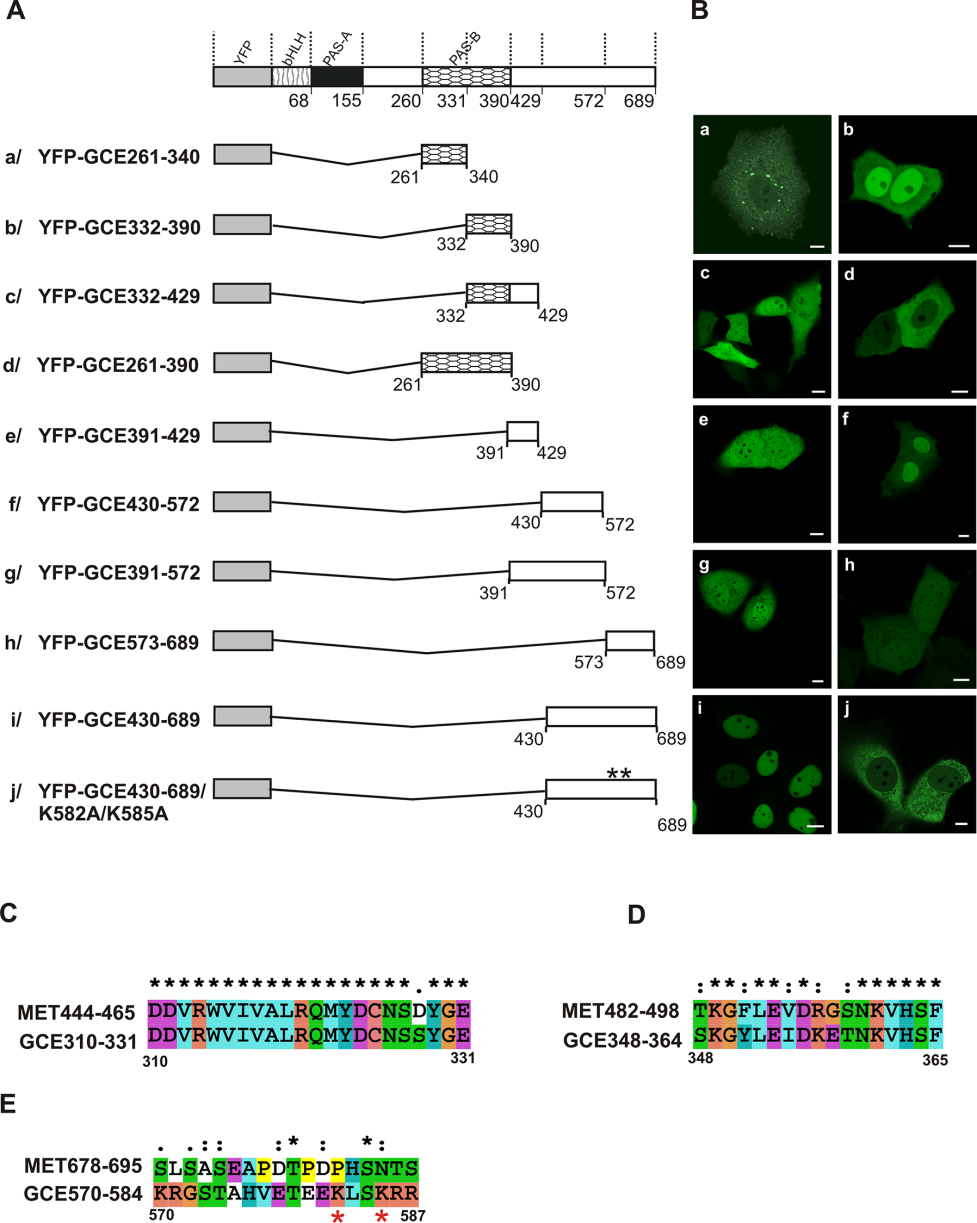
Subcellular distribution of the GCE mutants. Experiments identifying NLS and NES motifs in GCE, using a series of deletion mutants tagged with YFP revealed differentiated localization of truncated proteins. The subcellular localization of the expressed proteins was analyzed by confocal microscopy 20-24h after the transfection of COS-7 cells. (A) Schematic representation of the GCE derivatives. Regions of GCE are depicted using different patterns. The length of each domain in the diagram is arbitrary. (B) Representative images (single confocal plane) of the typical subcellular distribution of the GCE derivatives. Bar, 10 μm. (C) ClustalX alignment of the MET444-465/GCE310-331 encompassing NES in PAS-B domain. (D) ClustalX alignment of the MET482-498/GCE348-364 encompassing NLS in PAS-B domain. (E) ClustalX alignment of the MET678-695/GCE570-584 encompassing NLS in C-terminal fragment of GCE. Asterisks indicate identical residues, and colons indicate similar residues. Residues numbered according to the sequence of GCE are given at the bottom of the alignment. Red asterisks indicate mutated residues.

We observed the distribution of full-length GCE (Fig 1Aa) in both the nucleus and cytoplasm of transfected cells (Fig 1Ba) similarly to the distribution of the YFP protein (Fig 1Ab) expressed as a control (Fig 1Bb). The N-terminal fragment of GCE that encompassed the bHLH region (Fig 1Ac) was primarily in the nucleus (Fig 1Bc). The alignment of the MET and GCE bHLH domains revealed not only the conservation of basic amino acids from MET in GCE, but also the presence of some additional basic amino acids in the GCE (Fig 1C). The predominantly nuclear localization could be linked to the interaction of basic residues with DNA. The PAS-A domain (Fig 1Ad) was localized in the cytoplasm (Fig 1Bd), which suggests the activity of NES signal identified for MET [33] despite a few amino acid residues in GCE that didn’t match (Fig 1D). The fusion of bHLH and the PAS-A domain (Fig 1Ae) was localized exclusively in the cytoplasm (Fig 1Be), which suggests that the bHLH domain contains no NLS signal, or a very weak one, and the NES from PAS-A is dominant. The region between the PAS-A and PAS-B domains (Fig 1Af) is localized in the nucleus (Fig 1Bf). The performed alignment of MET and GCE sequences revealed that there was a greater accumulation of basic residues in the area of GCE in comparison to MET, where no such activity was detected (Fig 1E). Prediction software did not find any NLS in this part of GCE; however, the fusion of this region with PAS-A (Fig 1Ag) showed that there was protein accumulated both in the nuclei and the cytoplasm of the analyzed cells (Fig 1Bg). This suggests that there is a comparable strength in the NES from PAS-A and the putative NLS from the area that links PAS-A and PAS-B. More detailed research is needed to explain the results that were obtained. The extension of the region linking PAS-A and PAS-B by the N-terminal part of PAS-B to the E331 residue (Fig 1Ah) didn’t change the localization, as seen by the fluorescence in the nucleus (Fig 1Bh), whereas the N-terminal part of the PAS-B domain alone was uniformly localized throughout the whole cell (data not presented). Both of these results were unexpected. The alignment of the MET and GCE sequences demonstrated the presence of a sequence (see Fig 2C) previously documented as the active NES in PAS-B of MET [33]. However, the NES activity was not visible until the C-terminus of the N-terminal part of PAS-B was extended from the E331 to the N340 residue (Fig 2Aa). The extension led to a shift in the localization from ubiquitously distributed (data not shown) to being distributed in the cytoplasm (Fig 2Ba), which suggests that the presence of additional amino acids is required for NES activity.

The C-terminal part of the PAS-B domain (Fig 2Ab) showed that the localization was predominantly in the nucleus (Fig 2Bb), which is consistent with the previously documented presence of an NLS in this region in MET (Fig 2E) [33]. The expression of the C-terminal fragment of PAS-B extended by amino acid residues 391–429 (Fig 2Ac) resulted in a signal present both in the nucleus and the cytoplasm (Fig 2Bc), even though the NLS was in the C-terminal part of PAS-B. The lack of NLS activity under these conditions is likely due to additional amino acid residues which are post-translationally modified and which affect interaction with other proteins. It was recently shown that GCE residues 414–424 are responsible for the interaction with the nuclear receptor FTZ-F1 through the LIXXL motif [37]. Although there was an accumulation of PAS-B (Fig 2Ad) exclusively in the cytoplasm of the analyzed cells (Fig 2Bd), similarly to PAS-B of MET, the NLS activity was observed only after the mutation of the NES, which is dominant over the NLS in the absence of JH [33].

To study the localization of putative signals in the C-terminal part of GCE, this area was divided into three fragments: the N-terminal (residues 391–429), the internal fragment (residues 430–572) and the C-terminal (residues 573–689). The N-terminal fragment (Fig 2Ae) was localized both in the nucleus and the cytoplasm (Fig 2Be), while the internal fragment (Fig 2Af) shifted towards the nucleus (Fig 2Bf). However, the fusion of both fragments (YFP-GCE/391-572) (Fig 2Ag) resulted in uniform fluorescence throughout the cell (Fig 2Bg). The C-terminal fragment (Fig 1Ah) was localized both in the nucleus and the cytoplasm (Fig 2Bh), while the N-terminal extension of this fragment by residues in the internal fragment (YFP-GCE/430-689) (Fig 2Ai) redistributed fluorescence to the exclusively nuclear (Fig 2Bi). The *in silico* analysis identified two putative NLS’s in the analyzed area: a monopartite variant of the NLS at residues 582–589 and a bipartite NLS at residues 570–589. For activity of such signals the presence of basic amino acid residues like lysine or arginine is fundamental [31]. Intracellular localization of the derivative proteins after the scission between residues 572 and 573 is indirect proof of the existence of a bipartite NLS. In particular, the absence of basic residues K570 and R571 in the expressed truncated protein (Fig 2Ah and 2E) changed its distribution from exclusively nuclear to nuclear and cytoplasmic (Fig 2Bh). The final proof of the existence of an NLS in this area was obtained with the construct YFP-GCE/430-689, in which two amino acid residues (K582 and K585) from basic residues potentially crucial for NLS activity were substituted by A (Fig 2Aj). The mutations that were introduced caused the redistribution of the fluorescence from the nucleus to the cytoplasm (Fig 2Bj). A comparison of the GCE NLS revealed no homology with MET (Fig 2E). Thus, the NLS in the C-terminal part of the protein is unique to GCE. The localization of an analogous fragment of MET was strictly in the cytoplasm [33]. Each localization experiment was repeated three times to ensure the reproducibility of the results. Additionally, we performed localization experiments for the GCE derivatives described above in HEK293 cells and got the same results (data not shown).

Full-length GCE localize in both compartments of analyzed cells, in contrast to MET, which was predominantly in the nucleus [33]. This is a result of different pattern of signals that regulate the localization of GCE. Despite the conservation of NES signals in the PAS-A and PAS-B domains and the NLS signal in the PAS-B domain, there are intrinsic differences between GCE and MET. In addition to the deficiency of JH-independent NLS activity which is dominant for MET [33], the presence of a strong bipartite NLS in the C-terminal part of GCE seems to be a crucial difference in the specific NLS/NES pattern of these two proteins.

### The NLS in the PAS-B domain needs the activity of the NLS in the C-terminal fragment for GCE transport to the nucleus

**Fig 3:**
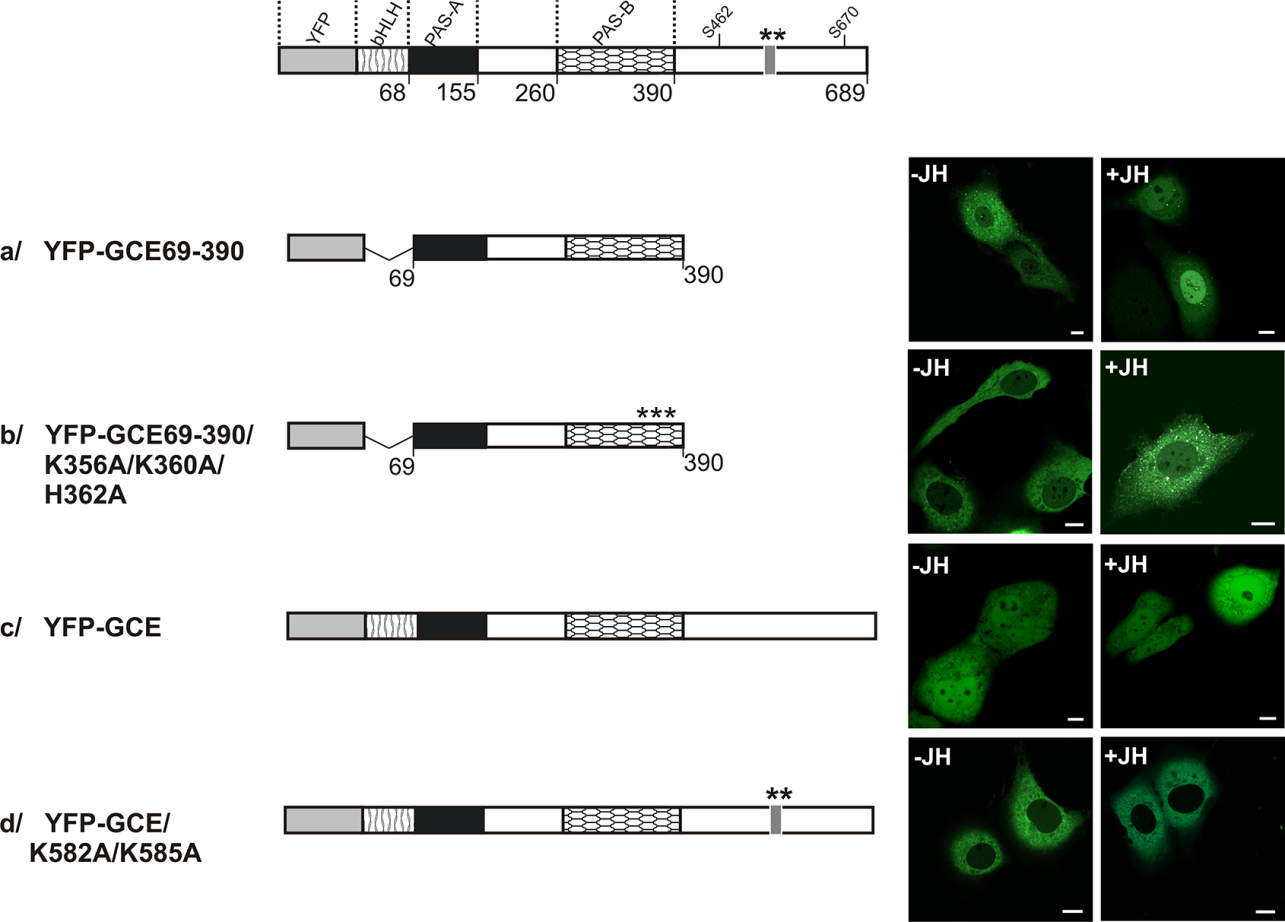
Subcellular localization of the NLS mutants. **(A)** The influence of JH on the subcellular distribution of the wild type and NLS point mutants of the bHLH-PAS-B area and full length GCE in COS-7 cells was analyzed. The subcellular localization of the expressed proteins tagged with YFP was analyzed by confocal microscopy 20-24h after the transfection of COS-7 cells in the absence or presence of JH. The length of each domain in protein schematic illustration is arbitrary. Regions of GCE are depicted using different patterns. Asterisks indicate mutated residues. Representative images (single confocal plane) present the typical subcellular distribution of the GCE derivatives. Bar, 10 μm. (B) ClustalX alignment of the MET588-631/GCE414-457 encompassing putative NES in C-terminal fragment of GCE.

Because of the high degree of homology between the MET and GCE PAS-B domains, we hypothesized that the PAS-B domain of GCE is also responsible for JH binding. Previously, we documented that the NLS activity in the PAS-B of *Drosophila* MET was dependent on the presence of JH [33]. Here, we tested the JH-dependence of the NLS activity in the PAS-B of GCE. As had been done previously for MET [33], the system was simplified by truncating the C-terminal fragment (residues 391–689) to avoid activating the C-terminal NLS, and the N-terminal fragment (residues 1–68) was truncated to prevent the bHLH domain from interacting with the DNA. Deletion mutants were created that encompassed the GCE residues from the PAS-A to PAS-B domains (residues 69–390). The resulting protein was accumulated in the cytoplasm in the absence of JH (Fig 3A), and the introduction of JH resulted in a shift to the nucleus (Fig 3A). To disrupt NLS in PAS-B domain, basic amino acid residues potentially crucial for NLS activity: K356, K360 and H362 were substituted by A. Mutant with an inactivated NLS was localized in the cytoplasm, both in the absence of JH and after adding JH (Fig 3B). This suggests that there was a loss of function and leads to the conclusion that NLS action in the PAS-B of GCE is JH-dependent similarly like NLS in the PAS-B domain of MET [33].

To further investigate the effect of JH on distribution of GCE we used the full length protein. Obtained results were unexpected. GCE localized both in the nucleus and cytoplasm independent on the absence or presence of JH (Fig 3C). Additional test was done to see the role of the NLS from the C-terminus of protein in transport of full-length GCE. An examination was made when the NLS activity in the C-terminal area was inhibited by introducing K582A and K585A mutations. In this case, localization was observed to be predominantly in the cytoplasm irrespective of the presence of JH (Fig 3D), suggesting that the NLS itself from PAS-B is insufficient for shuttling full-length GCE. Our results demonstrate that additional factor which is NLS in the C-terminal part of GCE is necessary for transferring of full-length GCE into the nucleus.

### Intracellular trafficking of GCE S462A and/or S670A mutants depends on the presence of JH

**Fig 4:**
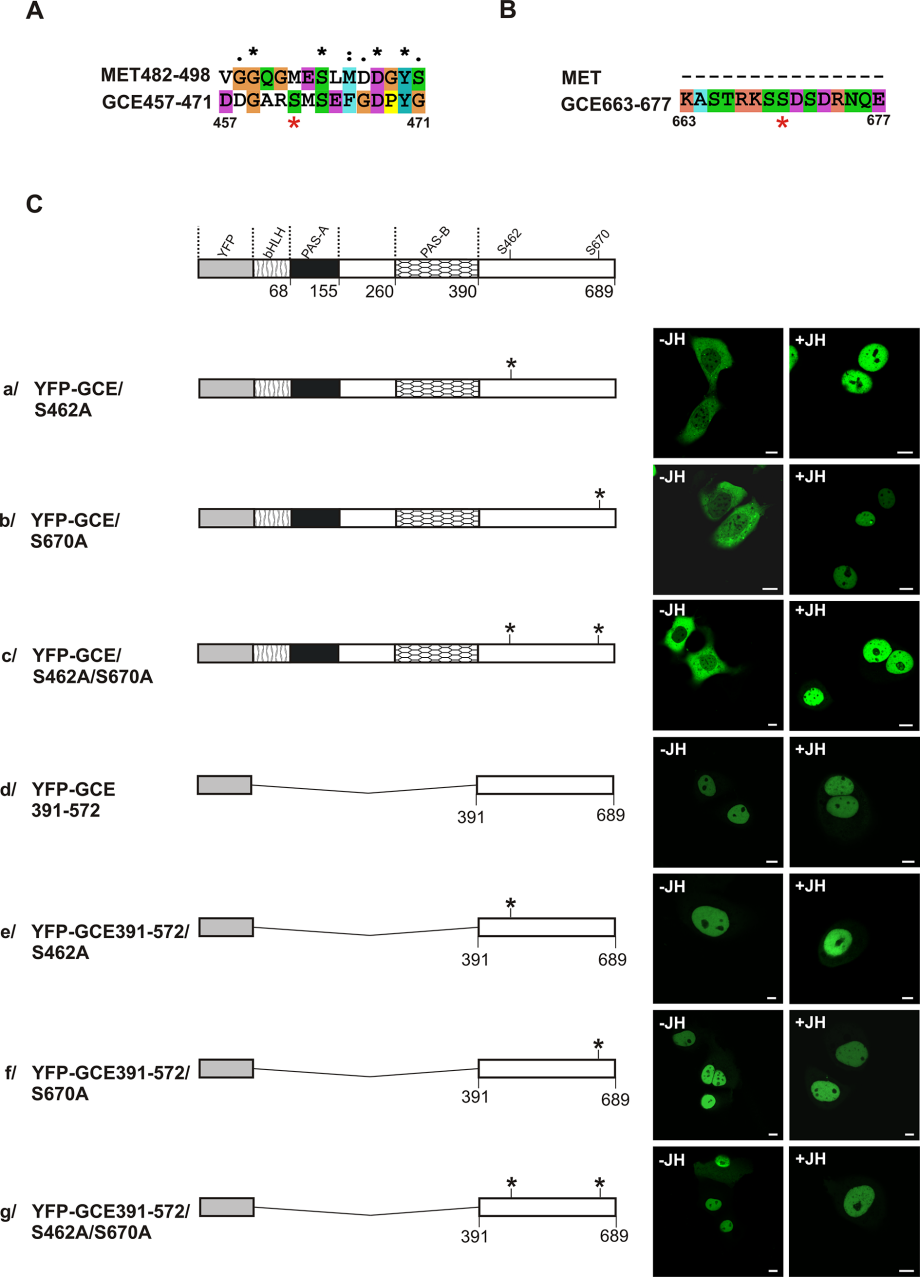
Subcellular distribution of the S462A and S670A mutants. (A) ClustalX alignment of the MET482-498/GCE457-471 encompassing GCE predicted 14-3-3 binding motif. (B) ClustalX alignment of the MET/GCE663-677 encompassing GCE predicted 14-3-3 binding motif. Asterisks indicate identical residues, and colons indicate similar residues. Residues numbered according to the sequence of GCE are given at the bottom of the alignment. Red asterisks indicate mutated residues. (C) The influence of JH on the subcellular distribution of the putative 14-3-3 binding sites mutants of the full length GCE and its C-terminal fragment was analyzed. The subcellular localization of the expressed proteins tagged with YFP was analyzed by confocal microscopy 20-24h after the transfection of COS-7 cells in the absence or presence of JH. The length of each domain in protein schematic illustration is arbitrary. Regions of GCE are depicted using different patterns. Asterisks indicate mutated residues. Representative images (single confocal plane) present the typical subcellular distribution of the GCE derivatives. Bar, 10 μm.

As indicated above, GCE is a paralog of MET, which is the putative juvenile hormone (JH) receptor [12]. It was documented that GCE binds JH with higher activity than MET [10]. Full-length GCE was localized both in the nucleus and the cytoplasm in the absence of JH and JH treatment had no effect on cellular distribution (Fig 3C). Since GCE is a transcription factor, it should be able to be transferred to the nucleus, so we decided to look for additional mediators of GCE shuttling. Using ELM, Scansite, Disphos 1.3 and NetPhos 2.0 predictors, the S462 and S670 were predicted with the highest scores to be phosphorylated and to be a part of the 14-3-3 protein binding motifs of GCE (Fig 4A and 4B). The ubiquitous family of 14-3-3 proteins is involved in the regulation of signal transduction by changing proteins subcellular localization [57]. A recent study described the influence on the specificity of signal transduction in the cell from the interaction of 14-3-3 proteins with partners that possess intrinsically disordered regions (IDR) [58], one of which we predicted in C-terminal fragment of GCE comprising S462 and S670 residues. Based on the knowledge that the 14-3-3 dimer usually interacts with a partner protein at two binding sites [59], two mutants were prepared of full-length GCE with single point mutations of the putative 14-3-3 binding residues: S462A or S670A (Fig 4Ca and 4Cb) and one mutant with substituted both predicted 14-3-3 binding residues GCE/S462A/S670A (Fig 4Cc). The mutants were N-terminally tagged with YFP, and their subcellular localization was analyzed. All the expressed mutant proteins were localized in the cytoplasm in the absence of JH (Fig 4Ca–4Cc). However, adding JH redistributed the mutants to the nucleus (Fig 4Ca–4Cc), which indicates that there is dependence on JH. These results suggest a possible role for 14-3-3 binding to GCE as modifying GCE localization in the absence and the presence of JH. The possible inhibition of 14-3-3 binding to GCE could be caused by the activity of other factors occurring in *Drosophila*, like de-phosphorylation by phosphatases or the masking of binding sites by other partners of 14-3-3 or GCE proteins. The lack of interaction with the mutant results in the unmasking of the NLS in the C-terminus of GCE. We hypothesize that in the absence of JH cytoplasmic localization of GCE mutants was the result of dominant activity of NES’s located in several domains. The presence of JH activates JH-dependent NLS in PAS-B which acts synergistically to unmasked NLS located in the C-terminal fragment of protein and transports GCE mutant to the nucleus. As previously mentioned, 14-3-3 typically binds as a dimer with two partner protein sites; interaction with a single site is also possible, although much weaker. Additionally, 14-3-3 can exist in a monomer form and perform chaperone-like activity [60,61]. It seems that in the case of GCE, neither of the two predicted 14-3-3 binding sites alone is able to continue interaction with these proteins, and both are equally important for putative binding function.

Previous studies documented that for JH binding by GCE the presence of PAS-B domain is necessarry and sufficient, and removal of GCE C-terminal region only lowers the protein yield [10]. However, in our study, we observed that the presence of JH influenced the localization of the mutants which had had a substitution of either one or both serine residues that had been predicted as sites that would be recognized by 14-3-3 and located in C-terminal fragment of GCE. For this reason, we decided to examine the JH role in localization of C-terminal fragment of GCE without ligand binding PAS-B domain. Both in the presence and absence of the hormone, our results showed that the full-length, C-terminal part of the wild-type GCE and all of the S462A, S670A mutants were distributed exclusively in the nucleus (Fig 4D–4G). Thus the influence of JH on the localization of GCE can not be mediated by the C-terminal domain without the presence of the ligand binding PAS-B domain and signals from both domains act synergistically.

**Fig 5:**
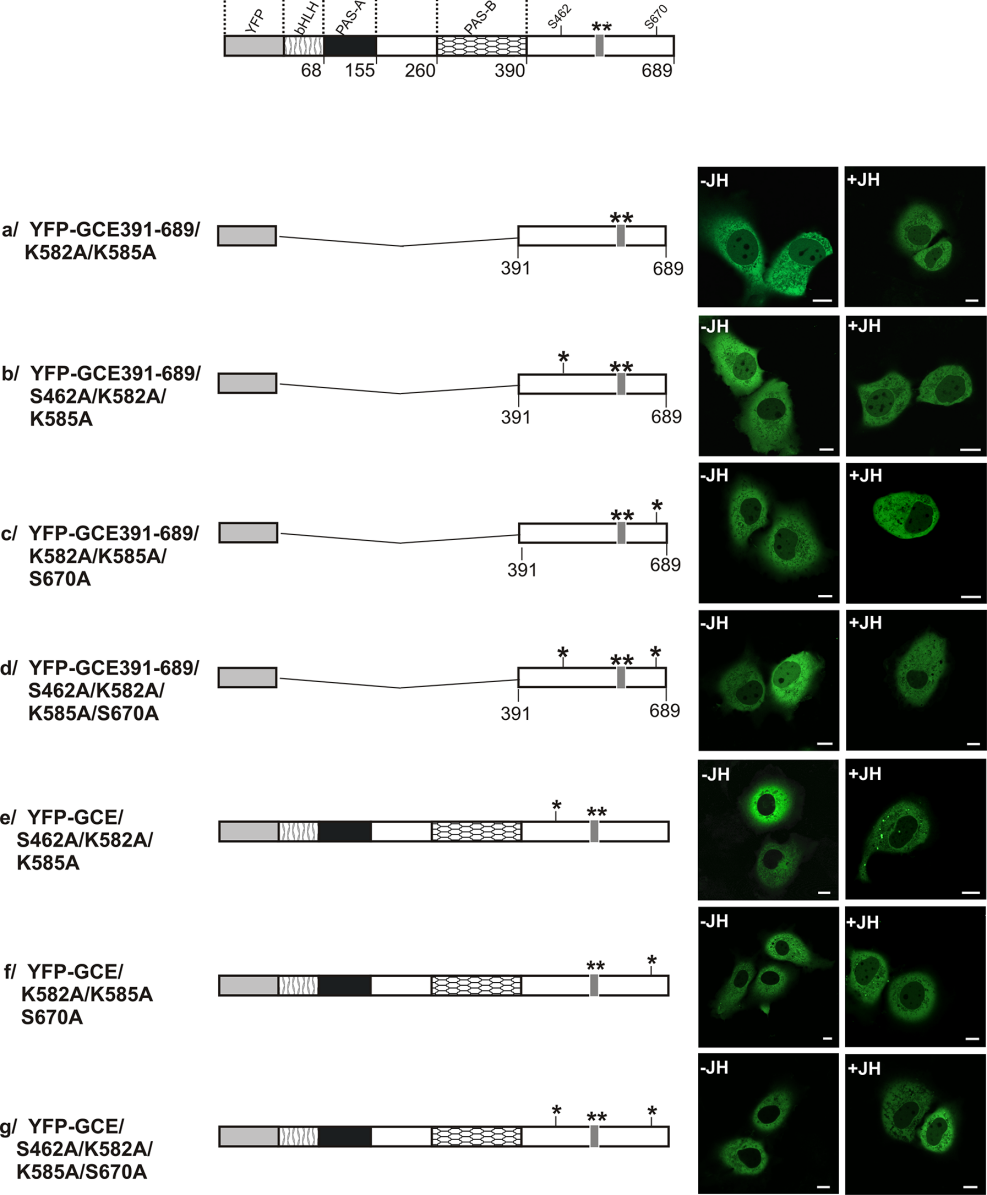
Subcellular distribution of the S462A and/or S670A GCE mutants with disabled C-terminal NLS. The influence of JH on the subcellular distribution of the S463A and/or S670A with deactivated NLS of mutants encompassing C-terminus or full length GCE was analysed. The subcellular localization of the expressed proteins tagged with YFP was analyzed by confocal microscopy 20-24h after the transfection of COS-7 cells in the absence and presence of JH. The length of each domain in protein schematic illustration is arbitrary. Regions of GCE are depicted using different patterns. Asterisks indicate mutated residues. Representative images (single confocal plane) present the typical subcellular distribution of the GCE derivatives. Bar, 10 μm.

Inactivation of NLS570-589 by K582A and K585A substitutions both in the C-terminal, wild-type fragment and in mutants where S had been substituted by A resulted in localization that was exclusively in the cytoplasm, independent of the presence of JH (Fig 5A–5D). Full length GCE mutants with inactive NLS in C-terminus localized exclusively in the cytoplasm both in the absence and in the presence of hormone (Fig 5E–5G) suggeting dominant role of the C-terminal putative NES after switching off dominant C-terminal NLS. Our results have shown that the activity of NLS 570–589 in the C-terminal part of GCE is crucial for nuclear localization of this protein.

## Discussion

**Fig 6:**
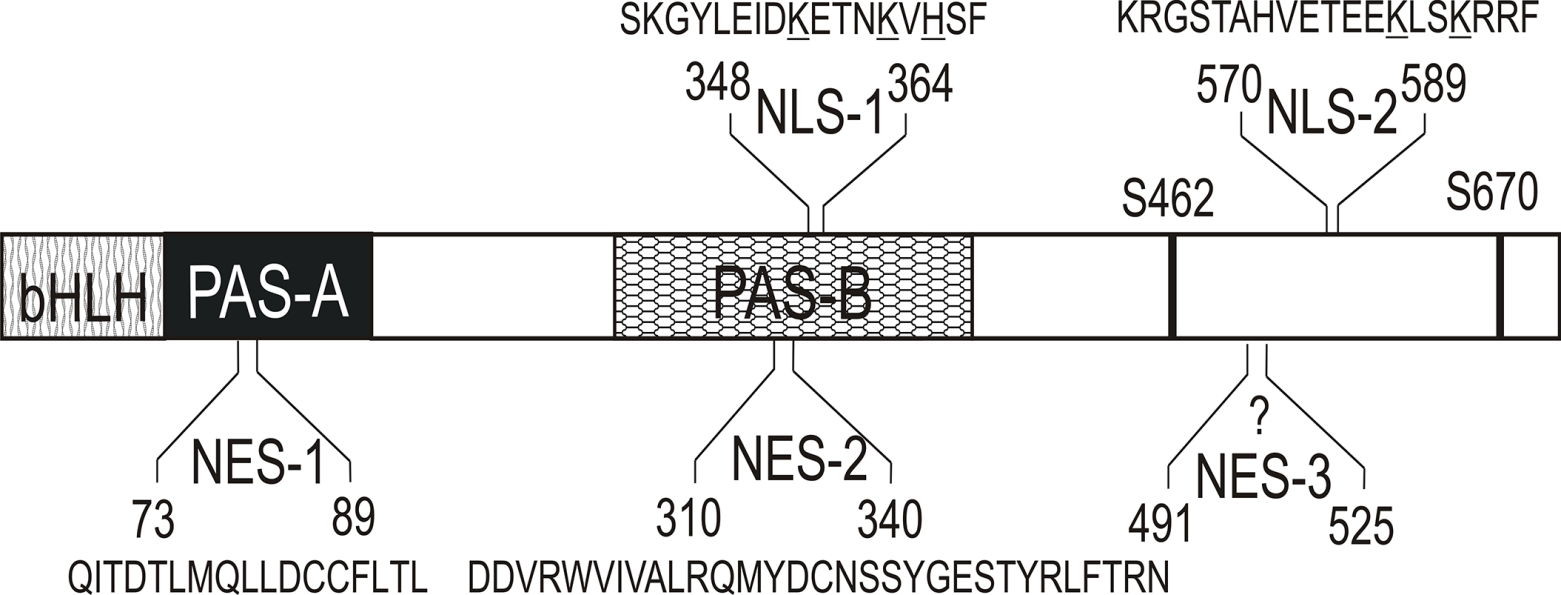
Schematic representation of NLS and NES signals residing within the GCE protein. Distribution of the NLS-1, NLS-2, NES-1, NES-2 and a putative NES-3 signals identified in the GCE protein. Specific domains of the GCE are marked with different graphic patterns according to Fig 1A. Residue numbering corresponding to the sequence of the GCE used in this study is given at the top and at the bottom of the sequences encompassing the NLS and NES signals.

**Fig 7:**
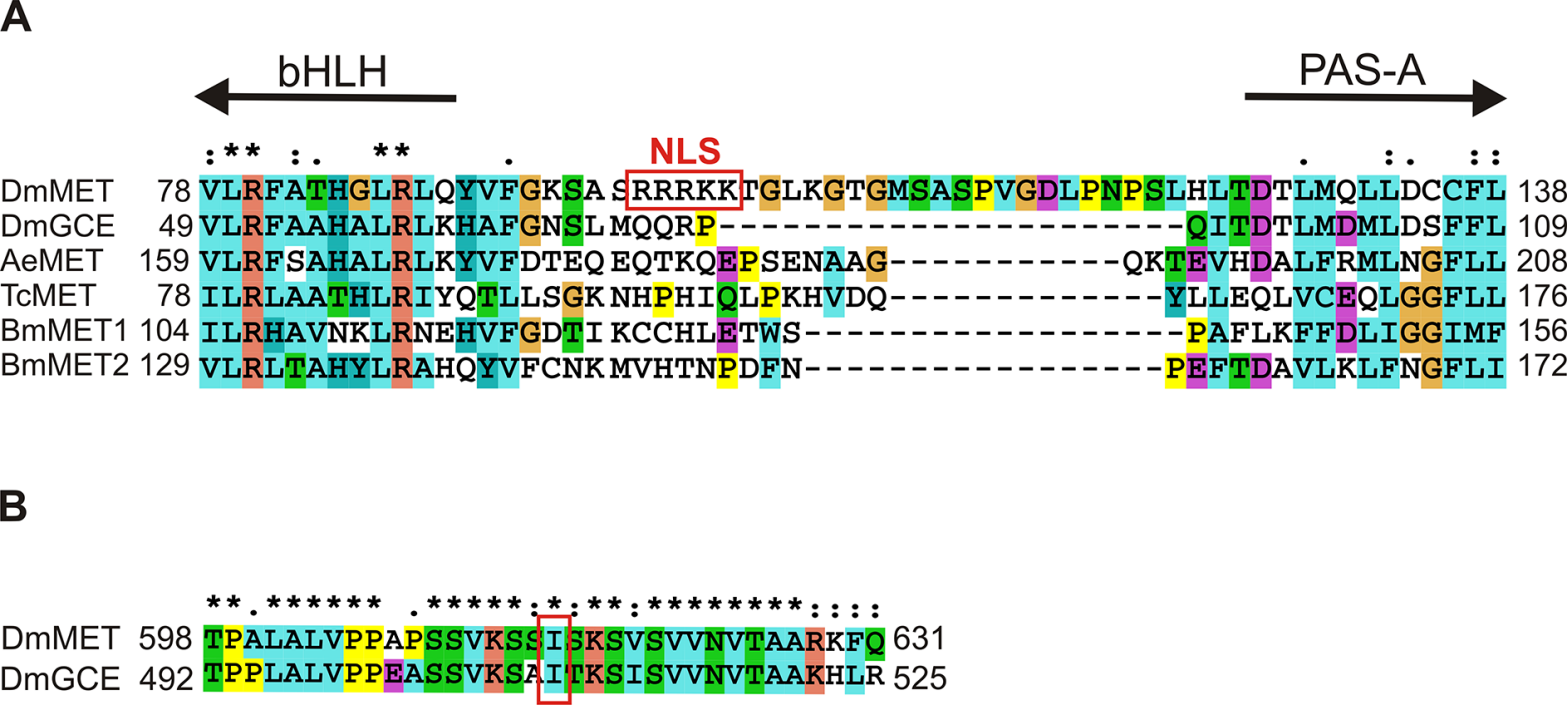
Alignment of the MET/GCE sequences. **(A)** Area that links bHLH and PAS-A domains from MET/GCE *Drosophila melanogaster*, MET *Aedes aegypti*, MET *Tribolium castaneum* and MET1/MET2 *Bombyx mori*. Red box indicate NLS unique for *Drosophila melanogaster*. (B) *Drosophila* MET598-631/GCE492-525 encompassing predicted NES. Red box indicate GCE residue I509 predicted as NES.

GCE is a known paralog of MET that belongs to the family of bHLH-PAS transcription factors in *D*. *melanogaster* [8,11]. Subcellular distribution is one of the key elements for the proper functioning of bHLH-PAS proteins [27]. This study has provided a detailed characterization of the NLS and NES signals which regulate the localization of GCE in cells. We have demonstrated that wild-type GCE is ubiquitously localized throughout the cell, in contrast to MET, which is localized predominantly in the nucleus [9,33]. The experiments verified the homology between GCE and MET with regard to their bHLH and PAS domains and verified the presence of active sequences equivalent to the signals in MET: NES-1 in PAS-A, NES-2 in PAS-B and a JH-dependent NLS in PAS-B (NLS-1) (Fig 6). There is an important difference which was detected in the short area that links bHLH and PAS-A, which in GCE lacks the dominant and ligand-independent NLS (RRRKK) that is present in MET [33]. A comparative analysis of homologous sequences in other insect species revealed that this signal is a unique feature of *D*. *melanogaster* MET (Fig 7A).

Many aspects of the functional control of bHLH-PAS proteins are analogous to those defined for nuclear receptor signaling [26]. Ubiquitous localization of transcription factor might be additional element of signaling pathways regulation. Human progesterone receptors (PR) are expressed from a single gene as two protein isoforms PR-A and PR-B [62]. Interestingly, PR-B which is localized both in the nucleus and in the cytoplasm of the cell is much stronger transcriptional activator than PR-A. PR-B additionally to classical nuclear genomic function mediates extra-nuclear non-genomic progestin activation of steroid receptor coactivator (Src)/mitogen-activated protein kinase (MAPK) signaling, whereas predominantly nuclear PR-A is not able to do so. Due to distinct cellular locations, progestin activation of Src/MAPK signaling can regulate selected target genes that lack direct PR binding response elements (PREs) [63,64]. In addition to regulation by ligand, activation of cellular signaling pathways can be sufficient to activate PR in the absence of hormone [63]. Lately, Liu *et al*. [4] suggested the presence of a non-genomic pathway in JH signaling. Additionally, the GCE/FTZ-F1 heterodimer, unlike MET/FTZ-F1, can activate transcription without methoprene [15].

It was previously shown that the overexpression of GCE in a tissue specific manner could partially make up for a lack of MET in resistance to pupal death or an eye defect but not for a defect in the male genitalia; thus, the functions of MET and GCE are only partially redundant and tissue specific *in vivo* [12]. Both GCE and MET interact with the nuclear receptor FTZ-F1 through the LIXXL sequence in their C-terminus, but the glutamine Q_R_ region in this area is a secondary receptor interaction site only for MET, which suggests that functional divergence can be attributed in part to regions located in the C-termini of MET and GCE [36].

The function of the C-terminal region, which shows the biggest sequence differentiation, is not known. In this paper we have shown that the C-terminal fragment of GCE is localized exclusively in the nucleus of the cell in contrast to the C-terminal fragment of MET, which was localized in the cytoplasm [33]. Additionally, we have identified the bipartite NLS 570-KRGSTAHVETEEKLSKRRF-589 (NLS-2; Fig 6) in this area. NLS-2 activity is necessary for the JH-dependent transport of GCE to the nucleus and contributes to the functioning of the JH-dependent NLS-1 in PAS-B. Our results shed light on the distinctive nature of the C-terminal parts of GCE and MET, which may be one of the reasons for the functional divergence of these two proteins.

Inactivation of NLS-2 residing in C-terminus of GCE leads to relocalization of this deletion mutant to cytoplasm. A search for clusters of hydrophobic residues (which are typical of NES) revealed one putative NES (NetNES ratio for I509 O.6) in the short region conserved both in C-terminus of GCE and MET (Fig 7B). Interestingly in our previous studies for MET we were not able to identify exact position of NES in C-terminus of this protein [33]. This sequence conservation between GCE and MET suggest similar role as putative NES-3 signal (Fig 6) in both proteins.

The ubiquitous family of 14-3-3 proteins is involved in the regulation of signal transduction [65]. Binding by 14-3-3 modulates enzyme activity, subcellular localization, structure, stability and molecular interactions of partner proteins [66]. 14-3-3 proteins usually recognize classical RSXpSXP, RX(Y/F)XpSXP or non-classical motifs and interact with phosphorylated serine residues [67–69], although 14-3-3 binding without phosphorylation was also reported [70]. 14-3-3 can function by changing the conformation of its partner, by functioning as a scaffold molecule to anchor proteins within close proximity of one another and acting as scaffold molecule that stimulates protein-protein interactions. 14-3-3 proteins are expressed in all eukaryotic cells and are highly conserved in amino acid sequences from yeast to mammals [71] what suggests that 14-3-3 might interact with GCE in COS-7 cells with the resultant effect on GCE localization. Mutants with an S462A and/or S670A substitution were localized in the cytoplasm and redistributed into the nucleus after the introduction of JH. The interaction between the 14-3-3 protein and its target can also be regulated by the presence of small molecule ligands [72]. We hypothesize that mutations of S462 and/or S670 disrupted the sequence recognized by the 14-3-3 proteins, mimicking the conditions in the cell when 14-3-3 is unable to bind GCE as a consequence of serine residue de-phosphorylation, competitive binding by other proteins or other unknown factors. The lack of such interaction would unmask putative NES-3, allowing GCE to be transported to cytoplasm in the absence of JH, while NLS-2 in cooperation with JH-dependent NLS-1 would transfer GCE to the nucleus under the influence of JH. The comparative analysis of MET and GCE sequences derived from selected insects species revealed the biggest differentiation of sequences in the C-terminal area of proteins [16]. This suggests that the C-terminal fragments of GCE and MET play an important role in protein functioning as specialized and differentiated paralogs in *D*. *melanogaster*.

Results presented in this paper, show combined impact of NLS and NES activities, the presence of JH and interaction partners on the GCE shuttling. We hypothesize that ubiquitous localization of the wild type GCE, which does not depend on JH, results from interaction of GCE with 14-3-3 proteins. This putative interaction is responsible for masking of NLS-2 and a NES-3 located in the C-terminus of the protein. The presence of JH alone is not sufficient for activation of GCE, as for inhibiting its interaction with 14-3-3 an additional not determined cofactor is required. When residues S462 and/or S670 (a putative 14-3-3 target) are mutated, signals located in the C-terminal fragment of GCE are dominant over other localization signals located in PAS-A and PAS-B domains. Synergistic action of NESs from different parts of GCE drives the full-length GCE mutated on S462 and/or S670 residues to cytoplasm in the absence of JH. However, in the presence of JH NLS-2 in cooperation with JH-dependent NLS-1 transfers GCE to the nucleus. S462, S670 GCE mutants are constitutively active.

## Conclusions

Our results show a very complex pattern of molecular elements which can direct GCE shuttling. It appears that GCE subcellular localization depends on the integrated action of several sequences and that their activity may be modulated by JH and partner proteins, like for example 14-3-3. Interestingly, the final localization of GCE seems to be regulated in a much more complex manner than it was observed previously for MET. This suggests the diverse and context-specific functioning of GCE and MET as transcription factors.
